# Sexual health behavior trends in a nationally representative sample of Canadian migrant adolescents from 2014 to 2022

**DOI:** 10.1186/s12889-025-23966-9

**Published:** 2025-09-02

**Authors:** Daniel Ji, Monica Rana, Mauricio Coronel-Villalobos, Nour Hammami, Elizabeth Saewyc

**Affiliations:** 1 Faculty of Social Work, University of Regina, 3737 Wascana Parkway, Regina, Saskatchewan S4S 0A2 Canada; 2 Stigma and Resilience Among Vulnerable Youth Centre, The University of British Columbia, University of British Columbia School of Nursing, T201-2211 Wesbrook Mall, Vancouver, BC V6T 2B5 Canada; 3 Child and Youth Studies, Trent University, Trent University Durham 55 Thoronton Road South, Oshawa, ON L1J 5Y1 Canada

**Keywords:** Migrant, Longitudinal, Reproductive health, Adolescent, STI prevention

## Abstract

**Background:**

Migrant youth in Canada are disproportionately vulnerable to the consequences of inadequate contraception use compared to their Canada-born peers, yet the sexual health behaviours of this population across time are poorly understood. This study mapped national Canadian trends in migrant adolescent sexual health behaviors disaggregated by migrant status and sex over eight years.

**Methods:**

Canadian Health Behaviour in School-aged Children (HBSC) study data were analyzed in 2014, 2018, and 2022 for sexual experience, condom, contraceptive pill, dual and neither method use at last intercourse. Age-adjusted logistic regressions examined prevalence trends of sexual health behaviors across years stratified by migrant status separately for boys and girls. Using non-migrants as referent group and 2014 as referent year, we then examined contrasts for disparities by year to test whether differences in sexual health behavior prevalence between migrant and non-migrant youth widened, narrowed, or remained stable for each sex.

**Results:**

Migrant girls were less likely to report sexual experience across all waves, a gap that remained consistent in 2018 and 2022. Compared to 2014, sexual health behaviors declined across all groups, especially migrants. Migrant boys were less likely to report sexual experience in 2018 and were less likely than Canada-born youth to use condoms in 2022. Contraceptive pill use was lower among migrant girls in 2022, this gap narrowed. Migrant boys were less likely to use contraceptive pills in 2018, this gap narrowed. Use of the dual method of contraception was lower in 2022 among migrant girls and boys. Migrant boys were more likely than non-migrants to use neither method in 2018 and 2022, this gap widened.

**Conclusions:**

Accessible contraception, culturally safe and relevant health information/services are recommended to counter declining contraception and safer sex behaviors among migrant youth.

## Background

Many people’s first sexual experience occurs during adolescence. Adolescent sexual health behaviors are evolving and complex. Even though the past two decades has seen a global increase in the use of contraceptives overall, condom use has declined [1, 2] and the prevalence of sexually transmitted infections among young people has increased [3]. Költő and colleagues [1] observed a decline in condom use from 2014 to 2022, with contraceptive pills being used by approximately one in four sexually active youths. Consequences like sexually transmitted infections (STIs) and unintended pregnancy [4] highlight the importance of safer sex health practices for adolescents. It is important for young people to have knowledge about sexual health behaviors to prevent sexually transmitted infections and unintended pregnancy. However, there are inequities regarding access to sexual health education and sexual health care between migrant and non-migrant youth [5, 6]. Many young migrants have never received basic sex education or preventive sexual healthcare [7]. Migrant youth have been found to experience barriers to accessing sexual education and healthcare including exclusionary practices in healthcare settings, lack of culturally sensitive services, and fears associated with anonymity breaches [8, 9]. Some migrant youth, depending on the social norms of their countries of origin, may be subject to cultural differences associated with premarital sex stigma, family expectations of abstinence, or stigma from community members [6]. Barriers to accessing sexual and reproductive health information and services for migrant youth constitute a significant public health concern. In this paper, we examine whether there are disparities in sexual health behaviors between migrant and non-migrant youth. The results of this study may point to the need for further in-depth assessment of the adolescent migrant experience in Canada.

Trends and disparities have been reported in past research on the sexual health behaviors of non-migrant youth. Saewyc et al. [10] examined time trends in sexual health behavior among boys and girls in British Columbia, Canada. The authors found from 1992 to 2003, youth were engaging in safer sexual health behaviors for both genders, these behaviors included fewer young people having sex at younger ages, and among sexually active adolescents responsible behaviors like contraceptive use and a declining trend in unintended pregnancies. A more recent study by Havaei et al. [11] reported that from 2002 to 2014, Canadian adolescent boys showed decreasing trends in sexual experience and increasing trends in the use of oral contraceptives. Use of the dual method (use of both condoms and birth control pills) among girls was stable from 2006 to 2014; however, an inverse trend was found among adolescent girls from 2002 to 2014 in their use of oral contraceptives and condoms, where they showed an increased reliance on oral contraceptives and less on condoms. Költő and colleagues [1] reported on the sexual health behaviors of adolescents internationally and reported that since 2014, condom use at last sexual intercourse declined among both boys and girls by 9 and 6 percentage points, respectively and that since 2018, approximately 30 percent of sexually active boys and girls did not use condoms nor contraceptive pills at last intercourse. They also reported socioeconomic differences in sexual health behaviors among boys, indicating that boys with high affluence were more likely to have ever had sex and more likely to have used a condom at last intercourse compared to boys with low affluence. All youth may be affected by trends of declining contraception use; however, migrant youth may be particularly affected due to disparities in family affluence [5, 12]. Migrant youth who experience economic stress have been found to have greater need for sexual and reproductive healthcare and experience more difficulties accessing care [5].

Even though adolescent boys and girls exhibit differences in trends of sexual health behaviors over time, there appear to be no studies to our knowledge that have documented disparities in the sexual behavior of migrant youth in larger national data, and whether there have been changes over time in such disparities which would warrant targeted efforts to address. Results based on regularly recurring prevalence studies are needed to effectively inform the development of interventions and policies to address migrant youths’ sexual health needs; however, few studies have documented migrant youth sexual behaviors over time at the national level.

### Study aim

In this paper, we seek to test whether disparities exist between migrant and non-migrant youth regarding sexual health behaviors. To this end, we examine trends and disparities in sexual health behaviors of migrant youth in Canada using a nationally representative sample. This study described nation-wide trends in the sexual health behaviors of migrant adolescents in Canada. Specifically, we addressed the following questions:

1. What are the changes in prevalence of time trends in sexual health behaviors in migrant and non-migrant boys and girls in Canada from 2014 to 2022?
2. Are there disparities in Canadian migrant adolescents’ sexual health behavior compared to their non-migrant counterparts in 2014, 2018, and 2022?
3. Are any gaps between migrant and non-migrant boys’ and girls’ sexual health behaviors narrowing, widening, or unchanged over time?

## Methods

### Design and setting

The Health Behaviour in School-aged Children (HBSC) study is an international cross-sectional study about the well-being of adolescents in their social context (i.e., at home and school, with family and friends) [13]. Data are collected every four years from schoolchildren enrolled in public schools aged 11 to 15 years old (in Canada, these ages correspond with grades 6 through 10) [14]. The survey is completed in English and French in Canada. Three levels of consent were required before students could participate in the HBSC study (i.e., school jurisdictions, school principals, then parental and/or student consent) as approved by ethics boards at the Public Health Agency of Canada (REB-2013–0022) and Queen’s University Research Ethics Board (6027003).

Each country or region follows a systematic, two-stage cluster sampling strategy using school classes as the primary sampling unit to ensure the sample is representative by age, sex and school type; details on data collection are described elsewhere [14]. Data are weighted such that responses from participants from each province contribute to the national results proportionally to the actual student population within that grade nationally [14]. In this study we analyzed data solely from the Canadian HBSC in 2014, 2018, and 2022 from youth in grades 9 and 10, because those below grade 9 were not asked about their sexual health behaviors.

As part of an ongoing study of migrant youth health, an advisory group of migrant youth from across Canada was convened, and they discussed analyses of the HBSC data at one of their virtual meetings. The purpose of the meetings was to review results with group members to contextualize the findings and benefit from feedback from migrant youth experiences to guide the formulation of recommendations or “next steps” to addressing any disparities observed in the results. The youth advisory group allowed us to obtain a more comprehensive understanding of migrant youth sexual health behaviors in Canada. Youth advisory members had migrated to Canada, were comfortable participating in English, and were between the ages of 16 and 24. The meetings took place online over Zoom with one researcher facilitating and two graduate research assistants taking notes. Advisory group members received an honorarium for their participation in the form of a $50 gift card.

### Measures

*Outcomes.* We measured five sexual health behaviors as outcome variables in this study. Sexual intercourse was measured by asking participants, “Have you ever had sexual intercourse (sometimes this is called “making love”, “having sex”, or “going all the way”)?” (yes/no). Participants who answered no to ever having had sexual intercourse were instructed to skip the additional questions on sexual health. The next four measures were contraceptive methods used at last sexual intercourse. We measured condom and birth control pill use by asking participants whether they used the method with response options of “yes”, “no”, or “I don’t know”. Participants who responded “I don’t know” were treated as missing. We measured use of the dual method (condoms and birth control pills) by identifying participants who answered “yes” to both items. Neither method was computed by identifying participants who answered “no” to both items.

*Predictor.* We measured migrant status using a dichotomized item asking whether participants were born in Canada. In 2018 and 2022 participants were asked “In which country were you born?” (Canada/Other country/I don’t know), whereas in 2014 participants were asked “How many years have you lived in Canada?” with the response options “I was born in Canada”, then four other options which included a range of years to select from. For this study, we created a “Born in Canada” variable by dichotomizing the responses to whether a participant reported whether they were born in Canada. Participants who did not report their migration status or responded “I don’t know” were excluded from analysis (*n* = 478)*.*

### Statistical analysis

We conducted a trends analysis on data from the Canadian HBSC study for three consecutive waves from school years 2014/15, 2018/19, and 2022/23 following Homma et al. [15]. Trends analysis uses logistic regression modeling to test for differences in the likelihood of outcome variables over time compared to a referent year, between groups compared to a reference group by year, and whether any observed differences between groups are widening, narrowing, or remaining consistent by including interaction terms in regression models (year by group). Thus, our trends analysis consisted of three steps: First, we examined sample demographics (i.e., age, sex, family affluence, and age of first sex) disaggregated by migrant status and survey year. We examined prevalence by year in outcome variables (whether a respondent had had sex, condom use at last sex, birth control pill use at last sex, and dual method and neither method at last sex), filtering by sexual experience. Next, we analyzed trends of prevalence of sexual health behaviors across years stratified by migrant status using age-adjusted logistic regressions, separately for boys and girls. Similarly, we examined contrasts for gaps across migrant status, stratified by year. Non-migrant youth comprised the reference group, and 2014 was the reference year. Finally, we examined whether differences in the prevalence of outcomes between migrant and non-migrant youth widened, narrowed, or remained stable for boys and girls from 2014 to 2018 and 2022 [15]. That is, we were interested in the relative change between the reference year and other years. We computed interaction terms (migrant status by survey year) in logistic regression models such that the full models for all outcome variables included migrant status, survey year, status-by-year interaction and age with non-migrant youth as the reference group for migrant status. In this analysis, a statistically significant interaction term suggests that the gap in recent rates of sexual health behaviors between migrant and non-migrant youth has significantly narrowed or widened over time. Consistent with interpretations provided by Homma et al. [15], Table 1 provides instruction for interpreting odds ratios for interaction terms. Age was used as a control variable in all regressions.

**Table 1:** Interpretation of odds ratios for gap analysis

Year	Original ORs	ORs for interaction terms	Outcome variable
2018, 2022	> 1	> 1	Widening
2018, 2022		< 1	Narrowing
2018, 2022	< 1	> 1	Narrowing
2018, 2022		< 1	Widening

We analyzed data using SPSS Complex Samples (Version 29) to account for the cluster sampling design and weighting of the data. Participants were excluded if they did not report their sex registered at birth or reported their sex at birth as neither male nor female (*n* = 358). Since sex was binary in 2014, this analysis used a binary measure for sex.

### Participants

Of the total 30,897 participants, 51.6% were girls and 48.4% were boys. Migrant youth comprised 16.9% of overall participants; 20.3% in 2014, 14.3% in 2018, and 14.3% in 2022. Migrant and non-migrant participants were compared using independent samples t-tests on age, family affluence, and age of first sex. Overall, migrant youth were older than their non-migrant peers, self-reported lower family affluence, and first had sex at a younger age (all significant at *p* < 0.05). We then investigated mean differences in demographic variables between migrant and non-migrant youth by year. Migrant youth were significantly older in 2018 and 2022 but not 2014. Migrant youth reported lower family affluence in 2014 and 2018, but the difference was not statistically significant in 2022. Table 2 displays prevalence estimates by year disaggregated by sex and migrant status.

**Table 2:** Demographic information by year for participants grades 9 and up

	2014 (*n* = 13,462)		2018 (*n* = 8323)		2022 (*n* = 8968)	
	*n*(%)	Mean(SE)	*n*(%)	Mean(SE)	*n*(%)	Mean(SE)
Age						
*Girls*	*Total* = 6869		*Total* = 4480		*Total* = 4582	
Migrant	1213 (17.7%)	15.37 (.05)	639 (14.3%)	***15.37 (.09)	623 (13.6%)	***15.42 (.06)
Non-migrant	5656 (82.3%)	15.38 (.05)	3841 (85.7%)	15.27 (.05)	3959 (86.4%)	15.25 (.05)
*Boys*	*Total* = 6739		*Total* = 3843		*Total* = 4384	
Migrant	1549 (23.0%)	15.42 (.05)	555 (14.4%)	***15.42 (.08)	655 (14.9%)	***15.42 (.07)
Non-migrant	5190 (77.0%)	15.41 (.05)	3288 (85.6%)	15.29 (.05)	3729 (85.1%)	15.29 (.05)
		Mean(SE)		Mean(SE)		Mean(SE)
Family affluence						
Migrant		***2.31 (.02)		***2.25 (.04)		2.45 (.01)
Non-migrant		2.43 (.01)		2.33 (.02)		2.40 (.03)

Data is from the HBSC study (Canada) from 2014–2022. Table reports weighted *n*’s and percentages. CI = confidence interval. *p <.05, **p <.01, ***p <.001

### Advisory group

Our youth advisory group is comprised of 21 members. The group consisted of 71% girls and 29% boys. All group members were first-generation migrant youth who had been in Canada for varying lengths of time from 1 year to over 10 years. Over half of the advisory group members had been in Canada for over 10 years (52.4%), under one-quarter (23.8%) had been in Canada for 5 to 10 years, and 23.8% had been in Canada between 1 and 5 years. In terms of country of origin, 38.1% were from India, 9.5% were from Pakistan, Syria, the Philippines, or Jordan respectively, and 4.8% were from Russia, South Korea, China, Zimbabwe, Syria, and Lybia, respectively.

## Results

To address our first research question, we examined the trends of prevalence of sexual health behaviors across years, stratified by migrant status separately for boys and girls. Table 3 displays the trends of adolescents’ sexual health behaviors across the three waves of data by migrant status. When comparing the same sub-group of youth over the years, girls were significantly less likely to have ever had sex in 2022, compared to 2014, this holds for migrant and non-migrant girls. Migrant boys were less likely to have ever had sex in 2018 compared to 2014, and non-migrant boys were less likely to have ever had sex in 2022 compared to 2014.

**Table 3:** Trends in sexual health behaviors (i.e., outcomes) between 2014 and 2022, by migrant status (%).^d^

				Trend comparison	
				2018^a^	2022^a^
	Prevalence comparison by survey cycle (%(n))			AOR^b^ (95% CI)	AOR^b^ (95% CI)
	2014	2018	2022		
Ever had sex					
*Girls*					
Migrant	14.1 (113)	9.2 (40)	8.7 (47)	.62 (.36, 1.07)	*.57 (.35,.94)
Non-migrant	23.2 (933)	20.6 (580)	16.0 (570)	.99 (.78, 1.27)	**.73 (.58,.91)
*Boys*					
Migrant	21.8 (224)	15.5 (62)	15.8 (82)	*.59 (.39,.88)	.66 (.42, 1.02)
Non-migrant	22.8 (842)	19.9 (487)	16.1 (507)	.89 (.73, 1.10)	***.70 (.57,.87)
^c^Condom use					
*Girls*					
Migrant	62.8 (63)	65.7 (23)	51.5 (20)	1.18 (.58, 2.38)	.65 (.34, 1.23)
Non-migrant	61.6 (559)	62.4 (343)	57.9 (313)	.96 (.69, 1.35)	.78 (.55, 1.11)
*Boys*					
Migrant	70.6 (137)	63.6 (30)	44.8 (33)	.74 (.35, 1.54)	**.34 (.16,.75)
Non-migrant	72.2 (568)	71.0 (314)	66.4 (314)	.96 (.66, 1.39)	.74 (.50, 1.09)
^c^Birth control pills					
*Girls*					
Migrant	57.7 (64)	35.6 (13)	10.9 (4)	**.35 (.17,.72)	***.08 (.03,.21)
Non-migrant	57.1 (521)	57.1 (319)	41.1 (213)	1.05 (.79, 1.39)	***.55 (.40,.77)
*Boys*					
Migrant	52.3 (88)	29.2 (13)	26.5 (16)	***.29 (.20,.42)	*.34 (.15,.78)
Non-migrant	54.7 (385)	59.7 (236)	46.2 (177)	1.25 (.83, 1.87)	.73 (.49, 1.08)
^c^Dual method					
*Girls*					
Migrant	34.4 (39)	9.5 (4)	9.4 (4)	-	**.19 (.07,.55)
Non-migrant	32.3 (298)	32.3 (183)	23.1 (121)	1.00 (.72, 1.39)	*.63 (.43,.93)
*Boys*					
Migrant	28.9 (62)	17.3 (9)	11.1 (8)	*.51 (.28,.94)	*.32 (.12,.83)
Non-migrant	33.1 (273)	33.3 (155)	26.9 (119)	1.04 (.71, 1.51)	.78 (.53, 1.14)
^c^Neither method					
*Girls*					
Migrant	13.9 (16)	8.3 (3)	39.4 (17)	.55 (.28, 1.10)	**4.04 (1.75, 9.32)
Non-migrant	13.8 (127)	13.3 (76)	27.8 (145)	.98 (.63, 1.54)	***2.47 (1.63, 3.72)
*Boys*					
Migrant	10.6 (23)	25.2 (13)	47.2 (33)	*2.74 (1.03, 7.31)	***7.46 (2.52, 22.09)
Non-migrant	12.7 (105)	10.7 (50)	22.0 (97)	.85 (.51, 1.39)	**1.98 (1.25, 3.14)

Data are weighted. ^a^Referent year is 2014. ^b^Odds ratios adjusted for age. ^c^Contraceptive methods were filtered by whether participant has had sexual intercourse. ^d^Filtered for whether responded has had sex and is in grade nine and above. aOR = adjusted odds ratio; CI = confidence interval. Cells not included are due to n < 5. *p <.05, **p <.01, ***p <.001

Condom use was significantly less likely among migrant boys in 2022 compared to 2014, but no differences were observed among their non-migrant counterparts. The likelihood of using contraceptive pills among migrant girls dropped significantly in both 2018 and 2022 compared to 2014, but only in 2022 among their non-migrant peers. Contraceptive pill use among migrant boys dropped significantly in 2018 and 2022 compared to 2014, but there were no significant increases or decreases from 2014 among non-migrant boys.

Use of the dual method among migrant boys was also highest in 2014, dropping significantly in both 2018 and 2022, compared to 2014. Dual method use among non-migrant boys dropped, though not significantly, in 2022 compared to 2014. Use of the dual method among non-migrant girls fell significantly in 2022 compared to 2014. For non-migrant girls, there was a significant decline in dual method use in 2022 compared to 2014. It is important to note that results are not releasable for 2018 due to small sample size. Results from 2022 should be interpreted with caution because the cell size was under 10.

There were significant increases from 2014 in the use of neither condoms nor contraceptive pills at last sex among both migrant and non-migrant girls in 2022, a result mirrored by boys in the data. However, migrant boys’ use of neither method was also significantly greater in 2018 compared to 2014 as well.

Table 4 shows the odds ratios for all the sexual health behaviors (i.e., outcome variables) by year with comparisons by migrant status (non-migrant youth are the reference group). Migrant girls were consistently less likely to have ever had sex than non-migrant girls in 2014 (aOR, 0.52; 95% CI, 0.38 to 0.72), 2018 (aOR, 0.32; 95% CI, 0.19 to 0.53) and 2022 (aOR, 0.43; CI = 0.26 to 0.69). Migrant boys were less likely than non-migrant boys to have ever had sex only in 2018 (aOR, 0.62; 95% CI, 0.42 to 0.90). Migrant boys were less likely than non-migrant boys to use condoms at last intercourse only in 2022 (aOR, 0.42; 95% CI, 0.21 to 0.86). Migrant girls were less likely than non-migrant girls to use contraceptive pills in 2022 (aOR, 0.16; 95% CI, 0.06 to 0.42). Partners of migrant boys were less likely than their Canada-born peers to use contraceptive pills in 2018 (aOR, 0.21; 95% CI, 0.10 to 0.45). Regarding use of the dual method, migrant boys and girls were both less likely than non-migrants to use both contraceptive methods in 2018 (girls aOR, 0.22; 95% CI, 0.12 to 0.41; boys aOR, 0.41; 95% CI, 0.20 to 0.84) and 2022 (girls aOR, 0.31; 95% CI, 0.11 to 0.88; boys aOR, 0.33; 95% CI, 0.11 to 0.98). Finally, migrant boys were significantly more likely to report no method used at last sex in 2018 (aOR, 2.75; 95% CI, 1.30 to 5.79) and 2022 (aOR, 3.24; 95% CI, 1.56 to 6.73).

**Table 4:** Odds ratios and 95% confidence intervals for all outcome variables by year (2014– 2022): comparisons by migrant status

	2014	2018	2022
	aOR^a^ (95% CI)	aOR^a^ (95% CI)	aOR^a^ (95% CI)
Ever had sex			
*Girls*			
Migrant^b^	***.52(.38,.72)	***.32(.19,.53)	***.43(.26,.69)
*Boys*			
Migrant^b^	.96(.73, 1.27)	*.62(.42,.90)	.89(.62, 1.28)
Condom use			
*Girls*			
Migrant^b^	1.00(.58, 1.72)	1.35(.45, 4.04)	.77(.39, 1.53)
*Boys*			
Migrant^b^	.92(.49, 1.73)	.70(.35, 1.41)	*.42(.21,.86)
Birth control pills			
*Girls*			
Migrant^b^	1.07(.58, 1.97)	.36(.13, 1.02)	***.16(.06,.42)
*Boys*			
Migrant^b^	.89(.51, 1.55)	***.21(.10,.45)	.40(.16, 1.02)
Dual method			
*Girls*			
Migrant^b^	1.11(.57, 2.16)	***.22(.12,.41)	*.31(.11,.88)
*Boys*			
Migrant^b^	.81(.46, 1.45)	*.41(.20,.84)	*.33(.11,.98)
Neither method			
*Girls*			
Migrant^b^	1.05(.56, 1.99)	.58(.24, 1.40)	1.74(.80, 3.80)
*Boys*			
Migrant^b^	.84(.32, 2.17)	**2.75(1.30, 5.79)	**3.24(1.56, 6.73)

Data are weighted. ^a^Odds ratios adjusted for age. ^b^Non-migrant is the reference group. aOR = adjusted odds ratio; CI = confidence interval. *p <.05, **p <.01, ***p <.001. Cells not included are due to n < 5

Table 5 shows the interaction effects by migrant status and year for all sexual health behaviors. Although gaps in the sexual health behaviors remained consistent in 2018 and 2022 among both migrant boys and migrant girls for sexual experience and condom use, there were notable changes in the use of contraceptive pills for migrant girls and boys, and use of neither method among migrant boys. First, for contraceptive pill use among migrant and non-migrant girls, the gap narrowed in 2022 by more than six times. For migrant boys, the gap in contraceptive pill use between non-migrant boys in 2018 also narrowed by a factor of over 4. The gap between migrant and non-migrant girls and boys remained consistent when it comes to use of the dual method of contraception. Finally, the gap in the use of neither method between migrant and non-migrant boys in the use of no method is widening over time per 2022.

**Table 5:** Trends in outcomes: interactions by migrant status and year^a^

	Ever had sex	Condom use	Birth control pills	Dual method	Neither method
	aOR^b^ (95% CI)	aOR^b^ (95% CI)	aOR^b^ (95% CI)	aOR^b^ (95% CI)	aOR^b^ (95% CI)
*Girls*					
Migrant × 2022	1.21 (.68, 2.15)	1.18 (.46, 3.02)	**6.85 (2.07, 22.73)	3.24 (.88, 11.91)	.62 (.22, 1.80)
Migrant × 2018	1.68 (.94, 3.00)	.78 (.23, 2.60)	2.87 (.90, 9.17)	5.10 (.80, 32.26)	1.80 (.57, 5.71)
*Boys*					
Migrant × 2022	1.05 (.67, 1.66)	2.18 (.84, 5.68)	2.15 (.73, 6.33)	2.48 (.72, 8.55)	*.27 (.08,.87)
Migrant × 2018	1.54 (.97, 2.42)	1.31 (.49, 3.47)	**4.20 (1.59, 10.99)	2.02 (.76, 5.38)	.31 (.09, 1.06)

Data were weighted. ^a^Non-migrant and year 2014 are the reference groups. ^b^Adjusted model included migrant status, survey year, and age along with the migrant status × year interactions. aOR = adjusted odds ratio; CI = confidence interval. *p <.05, **p <.01, ***p <.001

## Discussion

The purpose of this study was to examine trends in sexual health behaviors of migrant and non-migrant adolescents across Canada from 2014 to 2022. Fewer young people reported they have had sex in 2022 compared to 8 years earlier, regardless of migrant status. However, among adolescents who reported that they had had sex, there has been a downward trend in contraception use, which is particularly pronounced for migrant adolescents, especially for birth control pills. Data indicating a decrease in the use of the dual method and an increase in the use of neither method since 2014 are concerning and may help to explain the increasing rates of STIs among people under 30 in Canada [16]. The findings suggest that even though young people are having sex less overall in 2022 compared to 2014, they are also less likely to protect themselves from STIs or unintended pregnancy when they do have sex, especially migrant adolescents.

Our findings on lower prevalence of the contraceptive methods among migrant youth and the declining trends in their use may reflect previous research’s findings that migrant youth in Canada experience barriers to sexual health education and care needs [17]. Other studies have documented migrant adolescents may not be aware of Canada’s confidentiality policies in sexual health services, which in turn, may affect contraception use [18]. Meherali et al. [18] emphasized structural barriers to sexual health care, including costs for service, as well as cultural barriers of not knowing how to broach the topic with parents or being too embarrassed to discuss with parents, both of which disproportionately affect migrant adolescents. Such barriers may lead to higher rates of unintended pregnancies. Access to sexual health resources for this population may be further complicated by financial constraints and geographical barriers as well as barriers associated with cultural, language, and religious/family views that view sexual education unfavorably. Our findings are consistent with research on the unmet contraceptive needs of immigrant and refugee women in Canada, who are known for facing higher rates of unintended pregnancy than their Canadian-born counterparts [19]. Cultural beliefs and limited access to services highlight the need for interventions that are culturally tailored and accessible for migrants.

Whereas previous studies of Canadian youth have emphasized migrant youth’s sexual health needs or sexual health behaviors of all youth, this study provides important information on the specific trends and disparities of sexual health behaviors of migrant versus non-migrant youth from 2014 to 2022. These results should be interpreted in light of the COVID-19 pandemic, especially a large drop in contraceptive use from 2018 to 2022. COVID-19 likely affected the sexual health behaviors of many migrant and non-migrant youth in Canada. Many young people may have experienced increased parental monitoring, reduced privacy, and reduced physical interaction with peers [20]. Stay-at-home guidelines, lockdowns and social distancing rules likely limited opportunities for sexual activity among adolescents [21]. At the same time, access to sexual and reproductive healthcare may have been hindered by the pandemic. Lindberg et al. [21] explain that the ability to obtain private and confidential care may have been disrupted due to sheltering in place with parents or guardians, potentially inhibiting the use of contraception. If young people and/or their parents lost earnings due to the pandemic, then limited finances may have also acted as a barrier to accessing contraception. COVID-19 related school closures may have prevented young people from accessing sex education since many young people receive such education through school. Although COVID-19 influenced the sexual health behaviors of adolescents in Canada overall, the differences we observed between migrant and non-migrant youth warranted further probing.

### Migrant youth advisory feedback

Members of the migrant youth advisory offered important insights on the data based on their personal experiences. When talking about the declining trends of contraceptive use, specifically birth control pills, young people shared their fears about the long-term side effects of birth control pills (e.g., hormonal imbalance, rumors about infertility). They advised that many young people are discouraged from using birth control pills because of information from popular social media sources, which show videos of individuals sharing the negative consequences they experienced following their use and such information from social media is very easily accessible. One youth described the process of accessing birth control pills as “scary”, even more than having sex.

Reasons for their apprehension included reluctance to have their doctor or others in their family or community discover they received a prescription, citing possible stigma from family and friends as a reason for not choosing birth control pills. Cultural and religious expectations as well as family influence were offered as explanations for the reduced use of contraceptives by migrant youth. Such reasons are aligned with prior findings that complex socio-ecological factors including cultured, gendered, and religious social norms, have been found to discourage communication about sex, safer sex practices, and birth control among migrants in Canada [22]. Advisory members recommended combating negative perceptions of birth control by providing migrant youth with factual, science-based information at school or online. Moving beyond socio-ecological factors, group members raised systemic concerns with the barriers posed to migrant youth by the healthcare system to accessing birth control pills. Some members cited limited availability of physicians and healthcare providers, and prescription requirements as reasons migrant youth are not accessing contraceptives. Even though there were concerns among advisory group members about birth control pills specifically, some members stated their preference for doctor-prescribed methods that were longer-acting like IUDs, implants, or injectables over birth control pills.

When discussing the disparities in contraceptive use between migrant and non-migrant youth, advisory group members expressed their concerns about gaps in the sex education migrant youth receive in school as a barrier to making safer sex decisions. Members pointed out the failure of Canadian sex education to reflect the diversity of cultural backgrounds of migrant families. Some members reported that traditional sex education was not relevant or congruent with the beliefs or worldviews of migrant youth, making it less likely to influence their decision-making. They agreed that it is important to challenge the stigma associated with contraceptive use among migrant youth by normalizing discussions about sexual health with families and in schools. To increase relevance, one member suggested the possibility of involving community religious leaders in discussions about sexual health and contraception with migrant families. Another concern raised by a group member was the timing of sex education curriculum in Canada for some migrant youth. This member shared that if a youth arrives in Canada and is enrolled in school after the sex education curriculum in their province has ended, then they may not receive sex education at all. Since provinces vary in when sex education is provided, and for many young people school is the primary source of sex education, migrant youth may be particularly vulnerable to missing out on learning how to have safer sex. Indeed, our finding of the narrowing gap between migrant and non-migrant girls’ use of birth control pills may reflect successful efforts from the school system and increased school-centered awareness.

### Limitations

This study had several limitations. First, methods of contraception included in the HBSC questionnaire are limited to condoms, birth control, and “any other method(s)”. As such, the questionnaire may miss less common contraception methods. The 2018 version of the survey did not ask about other methods, and the 2022 survey included a “different type” without text specification. We suggest more options be included in a multiple-choice format for greater precision in analysis of sexual health behaviors (e.g., emergency contraception, implants, caps, spermicides). It would also be useful to know if young people are using non-medical methods (e.g., withdrawal). Given the sensitive nature of the subject, it is possible that people who are asked about their behaviors may either withhold information or respond in socially desirable ways, potentially skewing the data. This is of particular concern if young people’s cultural, religious or social context idealizes certain sets of responses associated with the values or norms specific to their context (e.g., not having sex outside of marriage or before a certain age). However, the anonymous survey method may help youth feel safe to respond honestly. There are likely important differences in trends of sexual health behaviors among individuals depending on religious affiliation and cultural background that research should examine in the future. Lastly, there is a lack of clarity to the question “Have you ever had sexual intercourse?” Its current wording does not adequately differentiate between penis in vagina sex, and anal or oral sex when asking about sexual experience. There is also no ability with this question to differentiate between consensual and non-consensual sex. HBSC does not currently ask about forced sex, which constitutes a limitation of the data. However, it is also important to note that since HBSC surveys are about sexual activity among 15-year-olds in multiple countries, and most 15-year-olds are not sexually active, it is difficult to ask for more detailed information about type of sexual behavior and still receive permission from schools at the population level to be able to conduct the survey. [23]. 

### Strengths

The main strength of this study is the use of data that are population based, stratified by school and classroom. Students enrolled in public schools across the country were sampled systematically to ensure representation of the general population. Moreover, the data were weighted by grade, allowing for greater confidence when making inferences about young people across the country. The statistical methodology of trends/gap analysis is also a strength in that it allowed for making inferences about the widening or narrowing of gaps across eight years in sexual behaviors between migrant and Canadian-born youth.

### Implications and recommendations

Following a review of the trends analysis results, we asked our migrant youth advisory group for recommendations or ‘next steps’ that could be taken to address disparities in contraception use between migrant and non-migrant youth in Canada. Advisory members provided several important suggestions. First, our findings align with recommendations from previous studies on migrant youth in Canada that there needs to be sex education for migrant youth that is easily accessible and relevant to a broad range of cultural groups, to fill a gap in knowledge needs for this growing population. Health care providers should develop and implement culturally tailored sexual health services specifically for migrant youth. They need knowledge of STIs and HIV as well as information about transmission, prevention, pregnancy, family planning, contraceptive use and how to access and navigate healthcare systems delivered in a way that is culturally safe and confidential. A review by Salam and colleagues [24] on enhancing sexual and reproductive health for adolescents indicated that sexual health education, counseling, and providing free or reduced cost contraceptives are all effective strategies for increasing contraceptive use and reducing adolescent pregnancies. Our findings illustrate this need is growing more urgent, given trends of decreasing contraceptive use among migrant youth. Community stakeholders such as agencies providing settlement services to migrant youth and families, advisory groups of migrant youth, and community members should be consulted about how to better tailor education and interventions to fit each community’s needs. Such interventions must be informed by respect for cultural values and a deep understanding of the specific needs of groups who may be facing marginalization.

Given that the age of first sex among migrant youth tended to be younger overall, early education about sexual intercourse, safety, pregnancy, STI prevention, and access to care whenever possible could be particularly beneficial for this group. Healthcare providers could be more welcoming by refusing to frame adolescents who were not born in Canada as a group that is inherently at risk but instead frame them as active and agentic about their sexual health. Researchers have cautioned against the reductionist categorization of migrant youth as inherently “at risk” for negative sexual health outcomes, as such risk narratives overlook structural and institutional limitations and barriers to access faced by this population [25]. Instead, Odger et al. [25] have suggested that youth be viewed as agentic actors navigating a complex landscape of sexual health messages. Although the prevention of STIs and unwanted pregnancies are important goals, education programs should also incorporate multiple perspectives about sex including pleasure, intimacy, and healthy relationships. Without the development and implementation of effective and easily accessible sex education programs for migrant youth that are relevant and flexible to cultural needs, especially among girls not born in Canada, we can expect the worsening trends in contraception use among migrants will continue.

In light of our findings that sexually active migrant youth are less likely to utilize contraception compared to their Canadian-born counterparts, it is essential for policymakers and program developers to enhance the accessibility of contraceptive methods for migrant youth. One potential solution is to provide free access to contraceptives, such as condoms, specifically for migrant youth who encounter financial challenges or face barriers to healthcare resources. This approach will eliminate the financial obstacle, improve access to safe contraceptive options, and support young individuals from families with limited healthcare access. Finally, our report on the widening and narrowing of gaps in the sexual health behaviors of migrant youth could lead to further follow up research with migrant youth, possibly using mixed methods, to ascertain information about what factors may have been associated with gaps in contraception use during specific years to inform future interventions.

## Conclusion

This study mapped the sexual health behaviors of migrant youth in Canada over eight years (in 2014, 2018 and 2022) to examine trends in sexual experience and contraceptive use. Sexual experience prevalence dropped across the three waves, but so did use of contraception, especially for migrant youth. There was a concerning increase in no form of contraception being used among migrant and non-migrant adolescents in Canada, particularly among migrant boys. Based on the results of this study, we recommend the implementation of culturally relevant sexual health education for migrant youth as well as people experiencing financial difficulties. Moreover, interventions intended to increase the use of contraception by migrant youth should be culturally relevant and specific, and foster agency and confidential access to contraception.

## Data Availability

The data that support the findings of this study are available from the World Health Organization’s Health Behaviour in School-aged Children study but restrictions apply to the availability of these data, which were used under license for the current study, and so are not publicly available. Data are however available from the authors upon reasonable request and with permission of World Health Organization’s Health Behaviour in School-aged Children study.

## Abbreviations

HBSC: Health behaviour in school-aged children
STI: Sexually transmitted infection
OR: Odds ratio
aOR: Adjusted odds ratio
CI: Confidence interval
